# Epidemiological, therapeutic, and survival trends in malignant pleural mesothelioma: A review of the National Cancer Database

**DOI:** 10.1002/cam4.5915

**Published:** 2023-04-16

**Authors:** Patrick Bou‐Samra, Austin Chang, Feredun Azari, Gregory Kennedy, Alix Segil, Emily Guo, Melina Marmarelis, Corey Langer, Sunil Singhal

**Affiliations:** ^1^ University of Pennsylvania Perelman School of Medicine Pennsylvania Philadelphia USA; ^2^ Department of Thoracic Surgery University of Pennsylvania Perelman School of Medicine Pennsylvania Philadelphia USA; ^3^ Division of Hematology & Oncology, Department of Medicine University of Pennsylvania Perelman School of Medicine Pennsylvania Philadelphia USA

**Keywords:** incidence, malignant mesothelioma, risk factors, treatment

## Abstract

**Background:**

Malignant pleural mesothelioma (MPM) is an aggressive cancer of the cells lining the pleural cavity with a low overall incidence. The National Cancer Database (NCDB) released in August 2022 updated data that reflect the newest trends in MPM.

**Methods:**

The NCDB was queried for patients diagnosed with MPM between 2004 and 2020. Variables collected included demographics, tumor characteristics, and treatment. Student's *t‐*test and independent‐samples proportions test were used for means analysis. Survival was assessed by the Kaplan–Meier method using SPSS version 28.

**Results:**

A total of 41,074 patients were diagnosed with mesothelioma, with a steady incidence (0.25%) between 2004 and 2017. The mean age of diagnosis was 70 (SD 13). 73.2% of the patients were males, 69% had no comorbidities, and 93.3% were white. More patients were diagnosed at Stage 1 after 2008 (*p* < 0.001). Since 2010, there has been a significant increase in patients offered treatment with 73.9% receiving some therapy (*p* < 0.01): 50.5% received chemotherapy, 27.6% surgery, 8.6% radiation, and 5.4% immunotherapy. The median overall survival was 10.3 months from diagnosis [95% CI: 10.2–10.5]. Risk factors associated with 30‐day mortality from surgical intervention included age (OR = 1.02, *p* < 0.001), male gender (OR = 1.3, *p* = 0.03), poorly differentiated grade (OR = 2.1, *p* < 0.001), Stage 4 (OR = 1.4, *p* = 0014), and epithelioid histology (OR = 0.51, *p* = 0.03).

**Conclusion:**

The current management of MPM is based on stage and histologic subtype. Due to the small numbers of patients at most academic centers, the NCDB provides a robust dataset to draw upon broad data points in treatment discussions with patients.

## Graphical abstract

The recently released National Cancer Database (NCDB) database proved the most updated trends in the prevalence, management, and diagnosis of malignant mesothelioma. Due to the small numbers of patients at most academic centers, the NCDB provides a robust dataset to draw upon broad data points in treatment discussions with patients.

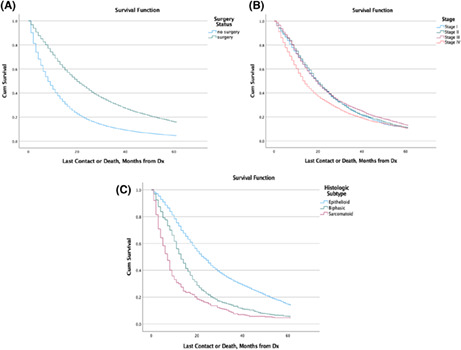

## 1 INTRODUCTION

Malignant pleural mesothelioma (MPM) is an aggressive cancer of the cells lining the pleural cavity. If left untreated, median survival is 4–12 months following diagnosis.^1^ Previous asbestos exposure is a risk factor associated with as much as 80% of cases.^2^ This exposure is believed to cause inflammation and free radical formation amounting in carcinogenesis of the pleura.^3^ Since the 1980s, regulations controlling asbestos use have led to the plateauing of MPM incidence.

Mesothelioma has three major subtypes: epithelioid, sarcomatoid, and biphasic. Surgery is the standard of care for resectable epithelioid MPM and has improved survival with a 5‐year survival of 10%–15%. Patients with sarcomatoid and biphasic histologies may not benefit from surgery.^4^, ^5^, ^6^, ^7^ The most common procedures to treat MPM are extrapleural pneumonectomy and pleurectomy/decortication, which has lower operative mortality. Surgery is often combined with radiotherapy and/or chemotherapy but a general consensus on a multimodal approach remains lacking.^8^ A prior dataset from the National Cancer Database (NCBD) with 20,988 patients showed that 22% of patients received chemotherapy alone, 13% received surgery alone, 2% received both surgery and chemotherapy, and 63% were observed. Overall survival (OS) was lowest in those who were just observed while median OS was greatest in those who received multimodal therapy including both surgery and chemotherapy.^9^

The aim of our study was to understand the most recent trends over the last two decades in incidence, histology, treatment, prognostic factors, and major differences in survival between patients with mesothelioma in the United States. Given the low incidence of this disease and insufficient data from any single academic center, we elected to utilize the NCDB in order to generate sufficient data for statistical significance. Identifying predictors of survival and factors associated with increased risk can help providers and patients in choosing their care options. This study is the first that uses the latest available data of the recently released NCDB public user files (PUF) 2020.

## 2 PATIENTS AND METHODS

### 2.1 Data source and inclusion criteria

Established in 1989, the NCDB is a nationwide, facility‐based, comprehensive clinical surveillance resource oncology database that currently captures 72% of all newly diagnosed malignancies in the United States annually.^10^ It is a joint project of the American Cancer Society and the Commission on Cancer of the American College of Surgeons. The American College of Surgeons has executed a Business Associate Agreement that includes a data use agreement with each of its Commission on Cancer‐accredited hospitals. The data used in this study are derived from a de‐identified NCDB file. The American College of Surgeons and the Commission on Cancer have not verified and are not responsible for the analytic or statistical methodology employed, or the conclusions drawn from the presented analysis of this data.

Patients included in our study were those with a history of pleural mesothelioma diagnosed between 2004 and 2020. All patients were 18 years or older and underwent treatment in a center designated by the Commission on Cancer. Patients with missing data were excluded from this study. Since this was an analysis of publicly available data, a waiver was granted for institutional review board revisions.

### 2.2 Variables analyzed and statistical analysis

The variables collected included patient demographics such as age at diagnosis, sex, race, insurance status, annual income, educational level, location in an urban versus rural setting, type of facility, and the Charlson–Deyo (CD) score. Other variables were related to tumor staging and treatment. They included histology, grade, type of treatment and staging defined according to the 6th Edition (2004–2009), 7th Edition Staging criteria (2010–2017), and 8th Edition Staging criteria (2018+). The outcomes that were analyzed included 30‐ and 90‐day mortality, and last contact or death months from diagnosis.

Two‐, 5‐year, and OS was assessed by the Kaplan–Meier method and life. An event was defined as patient death and patients were censored as to have survived if they were alive at the end of the studied interval. Each treatment method was sub‐analyzed by stratifying into NCDB analytic stage group and tumor histology. The log‐rank test was used for comparisons between groups. Two‐ and 5‐year follow‐up intervals from diagnosis were used to compare median survival between subgroups.

The log‐rank test was used for identifying predictors for 30‐, 90‐day, and OS. Clinically relevant variables and those who had a *p* < 0.2 on bivariate analysis were included in the final mode. Backward elimination was used to retain variables with a *p* < 0.05 significance value. The final model is reported as odds ratio (OR), 95% confidence interval, and *p* value.

## 3 RESULTS

### 3.1 Mesothelioma incidence and demographic trends

**FIGURE 1:**
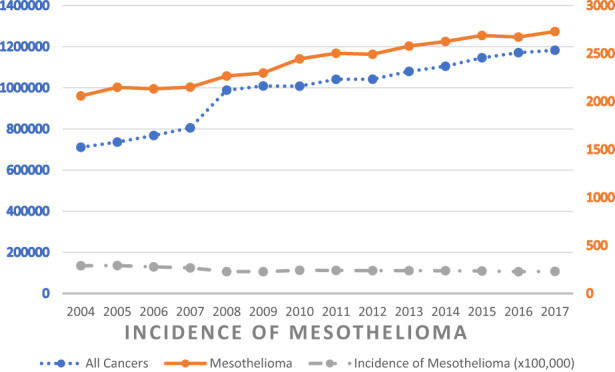
Incidence of mesothelioma between 2004 and 2017.

Over the 17 years evaluated, 41,074 patients were diagnosed with mesothelioma, with an average yearly 2417 new cases. The absolute number of patients newly diagnosed with mesothelioma is slightly increasing as seen in Figure 1. However, the incidence of mesothelioma compared to all cancers remained steady at 0.25% from 2004 to 2017 (Figure 1).

A total of 41,704 patients were included in our analysis. Mean age of diagnosis of patients with mesothelioma was 70 (SD 13) and median 73. It did not significantly change over the past 17 years (mean is 70.3 in 2004 and 70.7 in 2020; *p* = 0.3). The ages of patients ranged from 18 to 90. Most patients were between 71 and 80 (*n* = 13,759, 33.5%). 73.2% of the patients diagnosed were males compared to 26.8% females and that distribution was constant across the years. Most of the patients had no comorbidities, were White, and treated at a comprehensive community cancer program or academic/research program. Although most patients did not receive palliative care, the percentage of patients who were referred to palliative care increased from 8.8% in 2004 to 13.4% in 2020 (*p* < 0.001) (Table 1).

**TABLE 1:** Patient characteristics and demographics.

	Count (*N* = 41,074)	%
Age (years)		
<50	3349	8.2
Between 51 and 60	4449	10.8
Between 61 and 70	10,033	24.4
Between 71 and 80	13,759	33.5
>80	9484	23.1
Sex		
Male	30,059	73.2
Female	11,015	26.8
Race		
White	37,594	93.3
African American	2030	5.0
Asian	356	0.9
Other	315	0.8
Primary payor		
Not insured	803	2.0
Private insurance	10,918	26.9
Medicaid	1548	3.8
Medicare	26,403	65.1
Insurance status unknown	895	2.2
Median income quartiles 2000^a^		
<$30,000	3546	9.8
$30,000–$34,999	6043	16.8
$35,000–$45,999	10,262	28.5
≥$46,000	16,190	44.9
Percent no high school degree 2008–2012		
>21%	4965	13.4
13%–20.9%	9081	24.5
7%–12.9%	13,131	35.5
<7%	9857	26.6
Facility type		
Community Cancer program	2527	6.4
Comprehensive community cancer program	15,050	38.0
Academic/research program	14,434	36.4
Integrated network cancer program	7605	19.2
Urban/rural		
Metro	32,562	83.2
Urban	5839	14.9
Rural	754	1.9
Facility location^b^		
New England	2744	6.9
Middle Atlantic	7314	18.5
South Atlantic	7725	19.5
East North Central	7225	18.2
East South Central	2004	5.1
West North Central	2867	7.2
West South Central	3097	7.8
Mountain	1689	4.3
Pacific	4951	12.5
Charlson–Deyo score		
0	28,298	68.9
1	8440	20.5
2	2818	6.9
≥3	1518	3.7

^a^ missing 5033.

^b^ missing 1458.

### 3.2 Tumor diagnosis, histology, and staging

**FIGURE 2:**
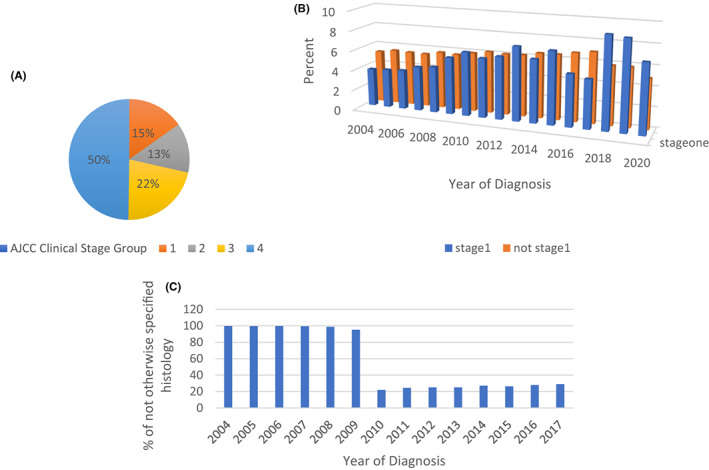
(A) Patient distribution according to AJCC Clinical Stage group. (B) Distribution of mesothelioma staging divided between Stage 1 and other stages between 2004 and 2020. (C) Percentage of patients without a specified histology based on year of diagnosis.

Most patients were Stage 4 on diagnosis (49.8%) (Figure 2A). There was a statistically significant increase in diagnosing patients while they were still in Stage 1 after 2008 from 13.5% to 15.6% (*p* < 0.001) (Figure 2B).

The most recorded histologic subtype is epithelioid (22.5%) with 53.5% of tumors without a histologic subtype specified. Prior to 2010, 98% of the data did not specify the histologic subtype compared to only 26% after 2010 (*p* < 0.001) (Figure 2C). Histology is considered a site specify factors in the NCDB. A lot of these factors were collected starting 2010 and hospital registrars often did not go back to data collected prior to 2010 to find the histologic subtype. Also, some of these points are sparse because the source of such information is usually laboratory reports that may sometimes be directly sent to the physician. As such, the hospital registrars would not have access to it unless it is picked up later.

### 3.3 Treatment

Since 2010, 73.9% of patients received treatment (Figure 2). Over the 10‐year period between 2010 and 2020, there was a significant increase in patients who were offered treatment from 71% to 76% (*p* < 0.01). The treatment modalities were surgical, chemotherapy, radiotherapy, and immunotherapy.

#### 3.3.1 Surgery

Thirty percent were offered surgery and 27.6% of patients underwent surgery. There were no statistically significant differences between the proportion of patients offered surgery in 2004 and 2020 (*p* = 0.45). The mean number of days patients stayed in the hospital post‐operation was 7.8 days (SD 10), with no statistically significant difference between 2004 and 2020 (*p* = 0.8).

#### 3.3.2 Radiation

8.6% of patients underwent radiation. There was a slight but significant increase in the proportion of patients who underwent radiation in 2020 compared to 2004 from 20% to 21% (*p* < 0.001). Radiation was administered with a mean of 113 (SD 113) days after diagnosis. It was administered for a mean of 60 days (SD 233).

#### 3.3.3 Systemic therapy

50.5% of patients received chemotherapy. Among those, 33.1% received the treatment perioperatively and the rest as definitive chemotherapy. There was a statistically significant increase in chemotherapy utilization from 56.8% in 2004 to 61.5% in 2020 (*p* = 0.002). There was also a statistically significant increase in the use of multimodal therapy from 30% in 2004 to 42% in 2020 (*p* < 0.001). On average, it was administered 55 (SD 48) days from diagnosis.

Only 5.4% received immunotherapy. There has been a significant increase in its utilization from 2004 to 2020 from 0.6% to 27% (*p* < 0.001).

## 4 OUTCOMES

### 4.1 Overall survival

The median OS of our entire cohort was 10.3 months from diagnosis [95% CI: 10.2–10.5]. There was a steady increase in the median survival in the patients diagnosed between 2016 and 2019 to 12.1 months [95% CI: 11.7–12.5] compared to those diagnosed between 2004 and 2007 with a median survival of 8.97 months [95% CI: 8.7–9.3] (Table 2).

**TABLE 2:** Percentage of 2‐ and 5‐year survival based on treatment with stratification of each treatment subgroup into stage and histology (*p*‐value in stage and histology is in reference to first element in subgroup).

	2‐year survival (%)	5‐year survival (%)	Median months	*p*‐Value
Surgery				
Yes	44	16	19.8 (19.5–20.3)	<0.001
No	18	5	7.9 (7.8–8.1)	
Stage				
I	39	11	19.2 (17.9–20.4)	
II	39	11	19.2 (17.9–20.5)	0.4
III	41	13	18.9 (17–19.7)	0.2
IV	31	11	13.1 (12.4–13.9)	<0.001
Histology				
Epithelioid	45	14	22.2 (21.1–23.3)	
Biphasic	22	5	12.4 (11.3–13.5)	<0.001
Sarcomatoid	15	4	6.4 (5.5–7.3)	<0.001
Chemotherapy				
Yes	29	8	14 (13.8‐14.3)	<0.001
No	20	7	6.01 (5.8–6.2)	
Surgery				
Yes	45	21	21.7 (21–22.3) <0.001	<0.001
No	15	5	11.4 (11.2–11.6)	
Stage				
I	29	7	15.5 (14.8–16.1)	
II	31	7	16.1 (15.3–16.9)	0.4
III	33	8	15.5 (15–16.1)	0.4
IV	21	5	11 (10.7–11.4)	<0.001
Histology				
Epithelioid	35	9	17.3 (16.7–17.8)	<0.001
Biphasic	17	4	11.8 (11.1–12.4)	
Sarcomatoid	8	2	6.6 (6.3–6.9)	<0.001
Radiotherapy				
Yes	26	9	12.2 (11.5–12.8)	<0.001
No	24	8	10.2 (10–10.4)	
Stage				
I	32	7	23.8 (21.2–26.5)	
II	36	11	27.9 (24.3–31.5)	0.2
III	35	11	27.8 (25.7–29.9)	0.1
IV	11	2	12.3 (11.4–13.3)	<0.001
Histology				
Epithelioid	40	12	28.8 (26.8–30.8)	
Sarcomatoid	20	4	18.3 (15.7–20.9)	<0.001
Biphasic	6	1	9.3 (7.9–10.8)	<0.001
Immunotherapy				
Yes	27	3	15.2 (14.4–16)	<0.001
No	24	8	10.1 (9.9–10.3)	
Stage				
I	28	1	17.1 (15–19.1)	
II	30	7	15.9 (12.9–19)	0.44
III	28	3	15.1 (13.3–16.8)	0.07
IV	24	2	14.1 (12.9–15.3)	<0.001
Histology				
Epithelioid	37	7	18.2 (16.5–19.8)	
Sarcomatoid	26	4	15.1 (11.7–18.5)	0.06
Biphasic	17	1	9.0 (7.3–10.7)	<0.001

### 4.2 Surgery and survival

Patients who had undergone surgery had a median survival of 19.8 months [95 CI: 19.2–20.3] compared to 7.9 months [95% CI: 7.8–8.1] in those who had not undergone surgery. (*p* < 0.001).

**FIGURE 3:**
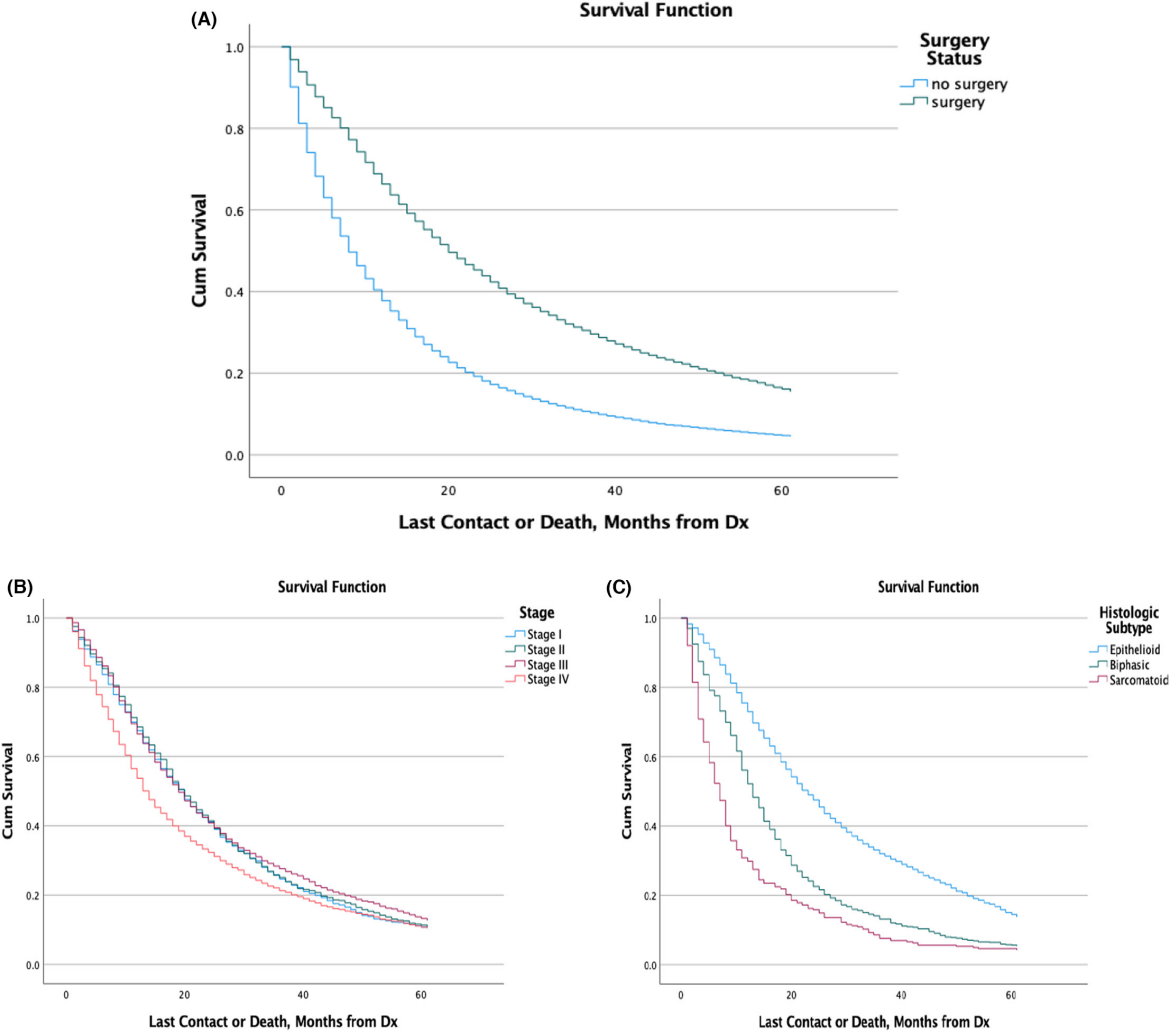
Survival curves based on surgery status (A). Survival curves among those who underwent surgery stratified by stage (B) or histologic subtype (C).

The 2‐year survival of those who underwent surgery was 44% compared to 18% in those who had not undergone surgery (*p* < 0.001). The 5‐year survival was 5% among those who did not undergo surgery compared to 16% among those who did (*p* < 0.001) (Figure 3A).

Among those who underwent surgery, those who were Stage I had a median survival of 19.2 months [95 CI: 17.9–20.3] which was significantly higher than those with Stage IV disease with a median survival of 13.1 months [95% CI: 12.4–13.9]. Stages I, II, and III had similar 2‐ and 5‐year survivals and were superior to Stage IV (*p* < 0.001) (Table 2; Figure 3B,C).

### 4.3 Systemic therapy and survival

#### 4.3.1 Chemotherapy

Those who underwent chemotherapy had a median survival of 14 months [95% CI: 13.8–14.3] compared to 6.01 months [95% CI: 5.8–6.2] among those who did not (*p* < 0.001).

**FIGURE 4:**
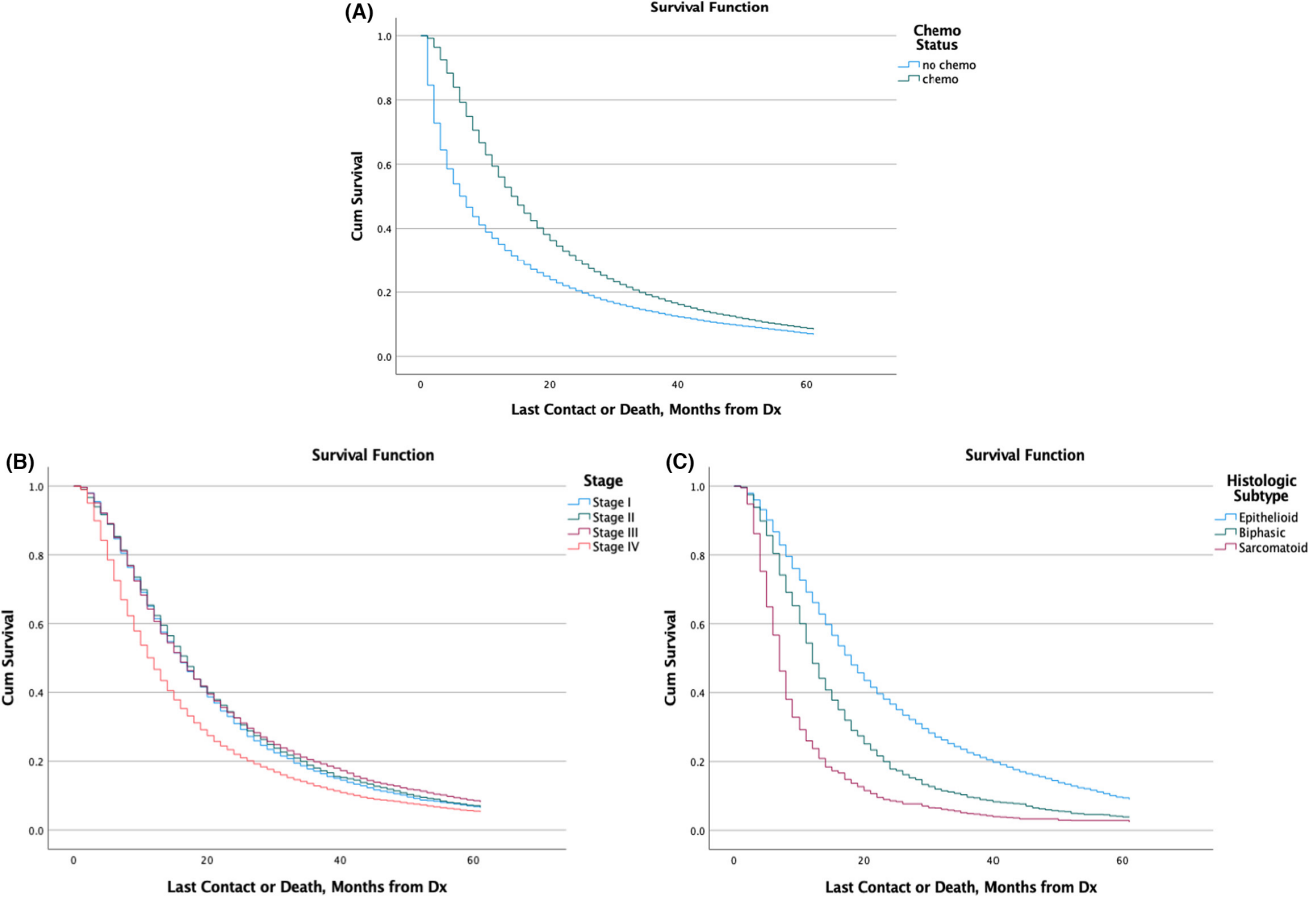
Survival curves based on chemotherapy status (A). Survival curves among those who received chemotherapy stratified by stage (B) or histologic subtype (C).

Among patients who had received chemotherapy, 35% of those with an epithelioid subtype survived for 2 years and 9% for 5 years. This was superior to both biphasic and sarcomatoid histologies (*p* < 0.001). When comparing stages among those who had received chemotherapy, patients with Stage 2 (*p* = 0.4) and Stage 3 (*p* = 0.2) had comparable 2‐ and 5‐ year survivals to Stage 1. However, those who were Stage 4 had a worse survival compared to all other stages (*p* < 0.001) (Table 2; Figure 4).

#### 4.3.2 Chemotherapy and surgery

We performed an additional subgroup analysis of patients who underwent chemotherapy. Those who had undergone surgery as well had a median survival of 21.7 months [95% CI: 21.0–22.3] compared to those who had not undergone surgery, 11.4 months [95% CI: 11.2–11.6]. The 2‐ and 5‐year survivals of those who underwent chemotherapy and surgery were 45% and 15% compared to 21% and 5% in those who did not undergo surgery, respectively (*p* < 0.001) (Table 2).

#### 4.3.3 Immunotherapy

Those who received immunotherapy had a median survival of 15.2 months [95% CI: 14.4–16] compared to 10.1 months [95% CI: 9.9–10.3] in those who had not received immunotherapy (*p* < 0.001).

Interestingly, 8% of patients had a 5‐year survival when they did not receive immunotherapy compared to 3% in those in those who received it. Meanwhile, the 2‐year percentage of patients who survived was 27% in those who had received immunotherapy compared to those who have not.

**FIGURE 5:**
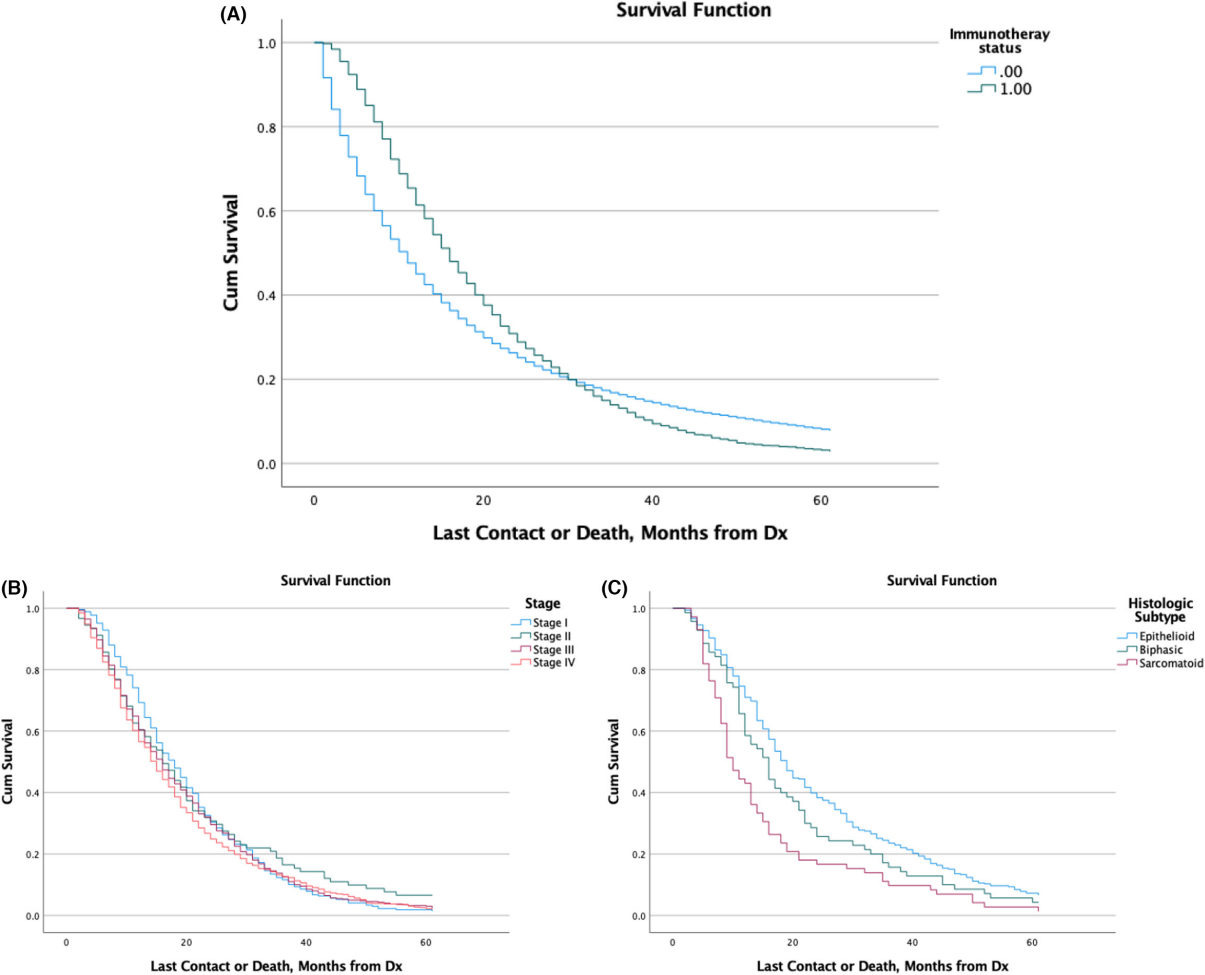
Survival curves based on immunotherapy status (A). Survival curves among those who underwent immunotherapy stratified by stage (B) or histologic subtype (C).

Among patients who had received chemotherapy, 37% of those with an epithelioid subtype survived for 2 years and 7% over 5 years. The results were comparable with biphasic (*p* = 0.06) but both were superior to sarcomatoid histology (*p* < 0.001). When comparing stages among those who had received surgery, patients with Stage 2 (*p* = 0.44) and Stage 3 (*p* = 0.07) had comparable 2‐ and 5‐year survivals to Stage 1. However, those who were Stage 4 had a worse survival compared to all other stages (*p* < 0.001) (Table 2; Figure 5).

### 4.4 Radiotherapy and survival

Those who received radiotherapy had a median survival of 12.2 months [95% CI: 11.5–12.8], which was superior to those who had not received radiotherapy with a median survival of 10.2 months [95% CI: 10–10.4] (*p* < 0.001).

**FIGURE 6:**
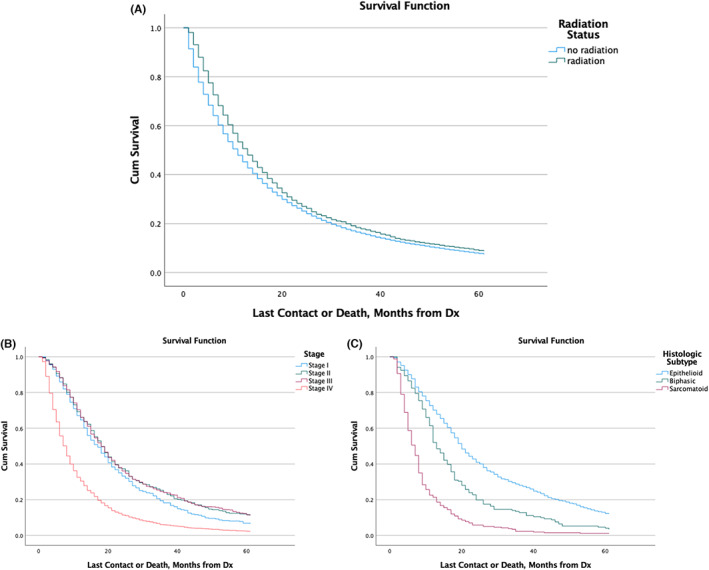
Survival curves based on radiotherapy status (A). Survival curves among those who underwent radiotherapy stratified by stage (B) or histologic subtype (C).

Among patients who had received chemotherapy, 35% of those with an epithelioid subtype survived for 2 years and 9% over 5 years. The results were superior to both with biphasic and sarcomatoid histologies (*p* < 0.001). When comparing stages among those who had received surgery, patients with Stage II (*p* = 0.2) and Stage III (*p* = 0.1) had comparable 2‐ and 5‐year percentage survivals to Stage I. However, those who were Stage IV had a worse percentage survival compared to all other stages (*p* < 0.001) (Table 2; Figure 6).

### 4.5 30‐ and 90‐day survival from surgery

Thirty‐day mortality correlated on a univariate level with mean age at diagnosis (OR = 1.05, *p* < 0.001), being female (OR = 0.7, *p* < 0.001), having a CD score of 1 (OR: 1.5, *p* < 0.001), receiving care at a community cancer network (OR = 0.55, *p* < 0.001), well‐differentiated grade (OR = 0.61, *p* = 0.02), Stage 1 disease (OR = 0.76, *p* = 0.1), epithelioid histology (OR = 0.354, *p* < 0.001), chemotherapy (OR = 0.13, *p* < 0.001), radiation (OR = 0.13, *p* < 0.001), and immunotherapy (OR = 0.21, *p* = 0.002).

On the multivariate level, age at diagnosis (OR = 1.02, *p* < 0.001), being male (OR = 1.3, *p* = 0.03), poorly differentiated grade (OR = 2.1, *p* < 0.001), Stage 4 (OR = 1.4, *p* = 0014), epithelioid histology (OR = 0.51, *p* = 0.03), treatment with chemotherapy (OR = 0.13, *p* < 0.001), and radiation treatment (OR = 0.17, *p* < 0.001) all correlated with 30‐day survival.

On the univariate level, 90‐day mortality correlated with mean age at diagnosis (OR = 1.05, *p* < 0.001), being male (OR = 1.4, *p* < 0.001), CD score of 1 (OR = 0.38, *p* < 0.001), receiving care at a community cancer center and integrated cancer network program (OR = 0.15 and OR = 0.61 respectively, *p* < 0.001), well‐differentiated grade (OR = 0.15, *p* < 0.001), Stage 1 disease (OR = 0.55, *p* < 0.001), the presence of malignant pleural effusion (OR = 1.3, *p* < 0.001), chemotherapy (OR = 0.2, *p* < 0.001), radiation (OR = 0.2, *p* < 0.001), and immunotherapy (OR = 0.25, *p* < 0.001).

On the multivariate level, age at diagnosis (OR = 1.02, *p* < 0.002), being male (OR: 1.22, *p* = 0.12), undifferentiated tumor grade (OR = 2.4, *p* < 0.001), Stage 1 (OR = 0.68, *p* = 0.002), the presence of a pleural effusion (OR = 0.58, *p* < 0.001), tumor histology as epithelioid (OR = 0.2, *p* < 0.001), having undergone radiation therapy (OR = 0.24, *p* < 0.001), having undergone chemotherapy (OR = 0.19, *p* < 0.001), and having undergone radiation therapy (OR = 0.2, *p* < 0.001).

## 5 DISCUSSION

Patients with MPM tend to be males, above the age of 60, and seek their care at academic/research programs or a comprehensive community cancer program. In term of prognostic factors, being male, having poorly differentiated disease, advanced stage, and non‐epithelioid histology all correlated with worse 30‐day, 90‐day, and overall mortality from surgery. The majority of patients have poor prognosis because of the late presentation and most patients are diagnosed with Stage 4 disease. However, there has been a trend to increased diagnosis of patients with Stage 1 disease resulting in an overall increase in survival over the same time span. We believe this is likely due to increased detection of pleural effusions and decisions to obtain pleural biopsies earlier in patient management.

The incidence of mesothelioma has not changed over the past 15 years. Indeed, we have much more effective screening and diagnostic mechanisms for most cancers and as such we are likely picking up more mesothelioma as well as more cancers in general, maintaining a steady ratio. On the other hand, this is a very indolent disease and median latency of 40 years and can be up to 50 years.^11^, ^12^ It was not until 1989 where the Environmental Protection Agency prohibited the use of asbestos in the United States with the bulk of its production during the 1970s.^13^ As such, the current numbers we are seeing are likely reflective of exposure 40–50 years ago before legislation was in place to control the use of asbestos, a very well‐known and documented risk factor.

In our study, OS for patients who underwent surgery alone was 19.8 months [95% CI: 19.5–20.3], which was superior to those who did not undergo surgery 7.9 months [95% CI: 7.8–8.1] (*p* < 0.001). Histologically, surgery is recommended only for the epithelioid subtype but may be considered for biphasic histology if the patient has early‐stage disease (“low volume disease”). This was reflected in our study with the significant increased survival of those with epithelioid subtypes who underwent surgery compared to those with other histologies. In our study, there were no significant differences between the proportion of patients offered surgery between 2004 and 2020. The surgical procedures offered are extra‐pleural pneumonectomy, extended pleurectomy decortication, pleurectomy decortication, and partial pleurectomy. Pleurectomy decortication and partial pleurectomy are debulking procedures which improve quality of life but do not necessarily prolong survival.^14^ The observed lack of increase in surgical therapy offered is likely related to the fact that there is no clear evidence‐based consensus on the surgical treatment of mesothelioma. The American Society of Clinical Oncology guidelines recommend maximal cytoreduction as part of multimodal treatment while the British Thoracic Society reserves the use of surgery for clinical trials.^15^, ^16^

In our study, chemotherapy has had increased utilization. This can be explained by the fact that chemotherapy is one of the only treatment modalities which confers a survival advantage, progression‐free survival, and response rate.^17^ Meanwhile, the use of radiation therapy has been steady with a slight but significant increase over time. It is not recommended for treatment alone but rather for more focal palliation and limit relapse in ipsilateral hemithorax.^16^ Timing of administration and combining it with chemotherapy should be addressed by a multidisciplinary team.^18^ It is noteworthy to mention that in our study, those patients who had received chemotherapy and surgery had a superior median survival to both chemotherapy or surgery alone further corroborating the importance of multimodality in the treatment of MPM.

Other determinants of the course of treatment rely on testing for biomarkers such as PDL‐1 (programmed cell death ligand 1) to determine the utility of immunotherapy. Over the past 10 years, there was an increase in patients who were offered treatment and a steady increase in survival. Traditionally, patient with a non‐epithelioid MPM have a worse prognosis and tend to be chemo‐resistant. Several studies suggest that the advent of immunotherapy can help address this subgroup as they have an increased expression of PDL‐1, the target of pembrolizumab.^19^ The non‐inferiority of survival in patients with sarcomatoid tumor might corroborate the role of immunotherapy in these tumors. We did note that those who underwent immunotherapy had an improved 2‐year survival, but that was not the case at 5 years. Although published after the release of the NCDB PUF, a Phase 3 clinical trial for unresectable mesothelioma showed that a novel dual immunotherapy regimen was associated with an improved survival with a median of 18 months in the treatment group compared to 14 months in the control group.^20^ This is in line with our 2‐year survival. Yet, the 5‐year survival of patients on immunotherapy appeared to be inferior to that of patients who were not on immunotherapy. Kartolo et al. evaluated patients with various cancer types who were started on immunotherapy, had complete response, and then had therapy discontinued. Some of these patients recurred and had a worse prognostic outcome and OS even when offered second‐line treatments.^21^ The NCDB does not list the time course of immunotherapy treatment which might shed more light on the observed trend. Also, immunotherapy is considered a novel therapy and will require long‐term follow‐up to understand it better. For instance, although a major improvement in cancer care, there are data that immunotherapy can cause hyper progression in a specific patient subgroup.^22^

This is a retrospective study, and it is hard to fully characterize the selection bias in the subgroups compared. Furthermore, the NCDB allows for analysis of rare tumors because of the large network it encompasses on a national level. It is heavily reliant on accurate reporting from hospital populations, which is not generalizable and representative of the entire population. It also does not break down surgery to the actual procedure performed for the treatment of MPM but rather into biopsy, debulking resection, en bloc resection, and biopsy. As such, granular analysis of the operations performed and a comparative study cannot be done.

In summary, the mainstay of management of MPM is through a multidisciplinary team and expert consensus based on stage and histologic subtype. Stage 1–3a and epithelioid histology are recommended surgical exploration with neoadjuvant or adjuvant chemotherapy. Otherwise, if the tumor is Stage 3 or 4 or unresectable, systemic therapy and/or supportive care are the current recommendation.^23^ The observed trends indicate increased use of chemotherapy and improved survival, but there are still no clear randomized control studies to dictate the best treatment options for this disease and this is partially because it is a rather rare disease. Larger multi‐institutional studies such as this one will help us better characterize this disease and eventually conduct collaborative randomized clinical trials to identify the best and eventually improved treatment modalities.

## AUTHOR CONTRIBUTIONS

Patrick Bou‐Samra: Writing‐original draft, conceptualization, methodology, formal analysis, data curation, project administration Austin Chang: conceptualization, methodology, data analysis, reviewing, editing Feredun Azari: conceptualization, project administration, reviewing, editing Gregory Kennedy: conceptualization, reviewing, editing Alix Segil: data curation, reviewing, editing Emily Guo: data curation, reviewing, editing Melina Marmarelis: conceptualization, reviewing, editing, supervision Corey Langer: methodology, reviewing, editing, supervision Sunil Singhal: conceptualization, data curation, original draft preparation, reviewing, editing, supervision

## CONFLICT OF INTEREST STATEMENT

There are no conflicts of interest or financial disclosures for any of the authors listed above.

## Data Availability

The data used in this study are derived from a de‐identified NCDB file. Patients included in our study were those with a history of pleural mesothelioma diagnosed between the years of 2004 through 2020. All patients were 18 years or older and underwent treatment in a center designated by the Commission on Cancer. Patients with missing data were excluded from this study. Since this was an analysis of publicly available data, a waiver was granted for Institutional Review Board (IRB) revisions.
